# Uneven selection pressure accelerating divergence of *Populus* and *Salix*

**DOI:** 10.1038/s41438-019-0121-y

**Published:** 2019-04-06

**Authors:** Jing Hou, Suyun Wei, Huixin Pan, Qiang Zhuge, Tongming Yin

**Affiliations:** The Key Laboratory for Cultivar Innovation and Germplasm Improvement for Salicaceae Species, College of Forestry, Nanjing Forestry University, Nanjing, 210037 China

**Keywords:** Evolutionary biology, Bioinformatics, Chromosomes, Evolutionary biology, Bioinformatics

## Abstract

*Populus* (poplars) and *Salix* (willows) are sister genera in the Salicaceae family that arise from a common tetraploid ancestor. The karyotypes of these two lineages are distinguished by two major interchromosomal and some minor intrachromosomal rearrangements, but which one is evolutionarily more primitive remains debatable. In this study, we compare the selection pressure acting on the paralogous genes resulting from salicoid duplication (PGRS) within and between the genomes of the two lineages. Purifying selection was determined to act more strongly on the PGRS in willow than on those in poplar, which would cause a faster loss of paralogous duplicates in willow. Therefore, *Salix* species are supposed to evolve faster than *Populus* species, which is consistent with the observation that the former are taxonomically and morphologically more diverse than the latter. In these two lineages, different autosomes were found to have been evolving into sex chromosomes. Examining the ω ratio and the PGRS in the sex determination regions in willow and poplar revealed higher convergent selection pressure and a faster loss of PGRS in the sex determination regions of both lineages. At the chromosome level, the sex chromosome in poplar is characterized by the lowest gene density among all chromosome members, while this feature is not observed on the sex chromosome in willow, suggesting that *Populus* species may inherit the more incipient sex chromosome from their progenitor. Taken together, *Salix* is supposed to be the nascent lineage arising from the additional round of genome reorganization that distinguishes the karyotypes of the two sister genera. In this study, assessment of ω ratios also detected a list of paralogous genes under unusual selection pressure, which could have special consequences for the adaptive evolution of Salicaceae species. In conclusion, the results of this study provide unique information for better understanding the genetic mechanism accelerating the divergence of these two closely related lineages.

## Genomics: Salicaceae family members under different selection pressures

Different selection pressure on duplicate genes explains how willow and poplar diverged from a common progenitor with four copies of their genome. A study by Tongming Yin and colleagues at Nanjing Forestry University in China compared the genomes of these two fast-growing trees to shed light on how they lost ancestral genes to retain only two copies of their genome. They found an uneven selection pressure on different genomic regions and a faster loss of duplicated genes in willow. This could explain why willow evolved more nascent and gave rise to over 300 species, whereas there are only about 29 species of poplar. Differences in gene density in the sex chromosomes are also key to the divergent evolution of these two sister lineages.

## Introduction

Whole genome duplication (WGD) is an important driving force in the evolutionary process of flowering plants^1,2^. Analyses of genomic data suggest that the extant angiosperm crown arises from a common paleohexaploid progenitor^3,4^. In addition to paleohexapolyploidization, one or more rounds of WGD recurred in the genomes of many angiosperms^5^, which simultaneously created thousands of paralogous gene pairs in the affected lineages. Population genetics predict that an entirely redundant duplicate copy cannot be maintained in the genome for a long time^6^. Following the ancient WGDs, some paralogs will be silenced and eventually eliminated, while many of the retained paralogs may be subject to changes in DNA sequence or gene expression, leading to sub or neo-functionalization^7–9^. Wholesale gene loss after WGDs can drastically shrink genome size and gene content, which has long been viewed as a critical driving force in the evolution of higher plants^10–12^.

*Populus* (poplar) and *Salix* (willow) are sister genera in the Salicaceae family. The two lineages are important fiber resources with rapid growth rates, ease of vegetative propagation, predisposition to hybridization, and high productivity of wood^13^. These characteristics, together with their relatively small genomes and the rapidly growing research resources, have led to Salicaceae species emerging as the model system for different aspects of genetic studies of woody plants. In the past decade, the whole genomes of poplar and willow have been sequenced and become publicly available^14,15^. Genomic analysis revealed that in addition to the paleohexaploidization shared in angiosperms, a lineage-specific WGD (known as salicoid duplication) recurred in the genome of the progenitor of these two lineages ca. 58~65 million years ago^14,15^. Genome comparison revealed that poplars and willows originate from a common paleotetraploid ancestor^16^. However, cytogenetic studies show that the extant poplars and willows mainly exist in diploid form^17^, suggesting that genome diploidization recurred after salicoid duplication. This process may accompany substantial genome reshuffling and gene losses^14,18–20^. Genome-wide comparison of sequences among different chromosome members suggested that after salicoid duplication, a series of reciprocal tandem terminal fusions of the duplicated chromosomes gave rise to the diploid progenitor of the modern taxa of these two lineages^14^. Approximately six million years later, two major interchromosomal rearrangements and several minor intrachromosomal rearrangements occurred subsequently^16,21^, which distinguished the karyotypes of willow and poplar. Previous studies have also revealed that primitive sex chromosomes evolve in poplar and willow, which are associated with different autosomes in the two lineages^21–23^. In addition to the divergences in genome structure and sex chromosome evolution, multiple lines of evidence, such as the 2 C DNA contents^24,25^, the k-mer estimation and the published genomes^14,15,26–28^, indicate that willows have smaller genomes and gene contents than poplars. Considering that these two lineages share a common ancestor, willows should lose DNA and genes at a faster rate than poplar after their divergence. However, the genetic mechanism triggering this scenario remains largely unknown.

The nonsynonymous (Ka) to synonymous (Ks) substitution rate ratio (ω = Ka/Ks) can be used as an estimator for selective pressure on DNA sequence evolution^29^. Using this analytical tool, Clark et al. detected an informative set of genes with significantly different patterns of substitution in humans different than that in chimpanzees and mouse among a total of 7,645 human-chimp-mouse orthologous genes^30^. With paralogous genes, this analytical tool is useful to navigate an evolutionary trajectory from an initial state of complete redundancy. By comparison of the selection pressure acting on paralogous genes with data from 26 bacterial, six archaeal, and seven eukaryotic genomes, Kondrashov et al. indicated that paralogs produced through duplication were subject to purifying selection, which would lead to losses of redundant genes^31^. In this paper, we assess the selection pressure on paralogous pairs resulting from salicoid duplication throughout the genomes of *Populus trichocarpa* and *Salix suchowensis*. We aim to detect whether there is uneven selection pressure accelerating the divergent evolution of these two sister genera.

## Results

### PGRS in poplar and willow

**Fig. 1:**
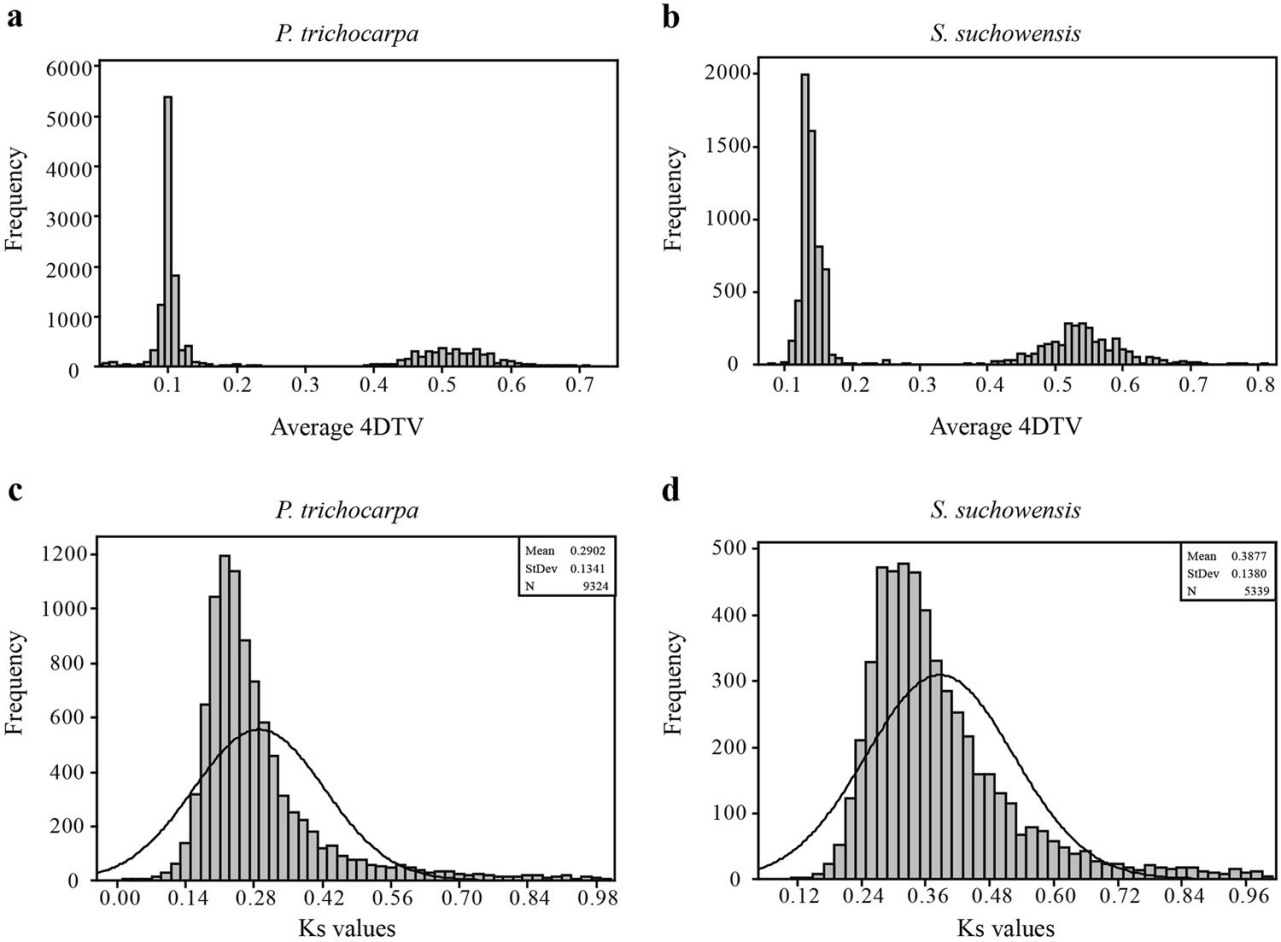
Plotting the average 4DTV and Ks values for paralogous genes on the syntenic segments (PGSS) in the genome of *P*. *trichocarpa and S. suchowensis* **a** Plotting the average 4DTV values for PGSS in the *P. trichocarpa* genome. **b** Plotting the average 4DTV values for PGSS in the *S. suchowensis* genome. **c** Plotting the Ks values for PGSS with 4DTV in the range of 0.050 to 0.150 in the *P. trichocarpa* genome. **d** Plotting the Ks values for PGSS with 4DTV in the range of 0.103 to 0.172 in the *S. suchowensis* genome

A total of 39,514 and 24,931 coding sequences contained in 19 chromosomal reconstructions were extracted from the genomic database of *P. trichocarpa* and *S. suchowensis*, respectively, and these genes were used to detect the PGRS in poplar and willow. Plotting the average 4DTV (four-fold degenerate site transversion) values for the paralogous genes contained on each syntenic segment revealed two peaks both in poplar and willow (Fig. 1a, b), and the highest peak was recognized to result from salicoid duplication according to Tuskan et al.’s study^14^. The highest peaks covered 4DTVs in the range of 0.0–0.2 in both lineages, which was consistent with the results in previous reports^14,32^. With a rejection significance *P* ≤ 0.01, the confidence interval [*μ*−2.58δ, μ + 2.58δ] would contain 99% of the variables covered by the peak associated with salicoid duplication. The confidence interval was [0.050, 0.150] and [0.103, 0.172] for *P. trichocarpa* and *S. suchowensis*, respectively. Syntenic segments with average 4DTV values outside of the confined ranges were subsequently filtered out in the following analyses due to concerns that they may not have arisen from the salicoid duplication. For the retained syntenic segments, we further calculated the Ks values for each paralogous pair. Based on the plotting of the derived Ks values (Figs. 1c, d), with 99% coverage, the paralogous pairs on syntenic segments with Ks values in the range of [0.000, 0.636] and those with Ks values in the range of [0.032, 0.744] (Supplementary Table S2) were recognized as PGRS in *P. trichocarpa* and *S. suchowensis*, respectively. According to Cui et al.’s study, the paralogous pairs with Ks values < 0.005 should be discarded to avoid fitting a component to infinity^3^; thus, we modify the range of Ks values for identifying PGRS in poplar as [0.005, 0.636] (Supplementary Table S1). In total, 8,991 and 5,161 PGRS were detected on the reconstructed chromosomes in *P. trichocarpa* and *S. suchowensis*, respectively (Table 1). The synteny for the detected PGRS among the poplar and willow chromosomes was separately shown in Supplementary Fig. S1 and Supplementary Fig. S2, respectively.

**Table 1:** Gene density, No. of PGRS, ω ratios range, mean ω ratio, and median ω ratio in poplar and willow genome

Chromosome	*P*. trichocarpa						S. suchowensis					
	Genes/Mb	No. of PGRS	PGRS/Mb	ω ratios range	Mean ω ratio	Median ω ratio	Genes/Mb	No. of PGRS	PGRS/Mb	ω ratios range	Mean ω ratio	Median ω ratio
I	93.7	2066	40.9	0.000–2.493	0.312	0.278	100.4	1000	38.0	0.000–0.904	0.273	0.253
II	104.5	1382	54.7	0.000–2.020	0.312	0.282	113.5	822	50.6	0.000–1.398	0.275	0.250
III	102.7	1122	51.4	0.000–2.493	0.311	0.273	114.2	710	58.0	0.000–1.177	0.281	0.255
IV	97.7	1020	42.0	0.000–2.114	0.303	0.275	106.0	605	45.1	0.009–0.943	0.261	0.249
V	100.6	1302	50.3	0.000–1.705	0.305	0.276	113.6	768	55.2	0.000–1.123	0.282	0.254
VI	100.8	1305	46.8	0.000–1.228	0.305	0.276	114.0	741	44.9	0.000–0.863	0.270	0.253
VII	94.4	635	40.7	0.000–1.282	0.304	0.272	103.8	314	38.1	0.000–0.859	0.279	0.252
VIII	116.4	1289	66.2	0.000–1.702	0.311	0.277	119.9	775	64.4	0.000–0.953	0.276	0.253
IX	131.8	892	68.9	0.000–2.114	0.304	0.274	122.4	518	54.0	0.000–0.943	0.263	0.251
X	113.2	1289	57.1	0.000–1.702	0.311	0.277	121.7	775	54.7	0.000–0.953	0.276	0.253
XI	91.9	700	37.8	0.008–1.779	0.315	0.279	91.2	357	32.2	0.008–0.904	0.276	0.248
XII	92.5	669	42.4	0.000–1.054	0.304	0.276	94.0	279	38.3	0.000–0.733	0.272	0.247
XIII	99.1	614	37.6	0.000–1.569	0.313	0.281	98.7	338	31.4	0.022–0.798	0.277	0.248
XIV	104.9	774	40.9	0.000–2.020	0.312	0.281	119.4	419	39.7	0.000–1.398	0.267	0.248
XV	97.2	656	42.9	0.000–1.054	0.301	0.274	110.8	386	44.4	0.000–0.763	0.267	0.239
XVI	99.5	677	46.7	0.000–1.228	0.304	0.277	121.4	710	53.7	0.000–1.177	0.281	0.255
XVII	96.6	530	33.0	0.013–1.353	0.318	0.292	98.5	270	31.6	0.009–0.749	0.268	0.249
XVIII	90.0	641	37.8	0.000–1.216	0.308	0.276	97.7	312	38.2	0.013–0.734	0.278	0.252
XIX	84.1	395	24.8	0.010–1.271	0.318	0.288	89.4	223	27.1	0.022–0.798	0.265	0.242
SDR	85.3	26	11.9	0.010–1.271	0.317	0.233	79.7	11	14.9	0.092–0.396	0.237	0.223
Genome wide	100.2	8991	22.8	0.000–2.493	0.309	0.277	108.8	5161	22.5	0.000–1.398	0.274	0.252

Gene coverages for the 19 chromosomal reconstructions were 98.0% and 93.7% of the total poplar and willow genomes, respectively^14,16^. Although most genes were assembled on chromosomes of poplar and willow, the reconstructed chromosomes are incomplete, and the integrity may vary among different chromosomes. Thus, the absolute numbers of genes are not comparable among different chromosomes. In contrast, gene density is a comparable parameter under such circumstances. As shown in Table 1, gene density ranged from 84.1/Mb (chromosome XIX) to 131.8/Mb (chromosome IX), and PGRS density ranged from 24.8/Mb (chromosome XIX) to 68.9/Mb (chromosome IX) among chromosomes in the poplar genome. In the willow genome, gene density varied from 89.4/Mb (chromosome XIX) to 122.4/Mb (chromosome IX), and PGRS density varied from 27.1/Mb (chromosome XIX) to 64.4/Mb (chromosome VIII). It is noteworthy that chromosome XIX, the sex chromosome in poplar, is characterized by the lowest gene and PGRS densities among all the chromosome members.

Correlation analysis shows that gene and PGRS densities on the corresponding chromosomes are highly correlated between poplar and willow, with a correlation coefficient equal to 0.79 (*P* = 0.000) and 0.89 (*P* = 0.000), respectively. The high correlation coefficients imply that loss of PGRS scales similarly across different chromosomes in the two lineages. On the reconstructed chromosomes, poplar is found to retain more PGRS than willow (8991 vs. 5161), indicating that willow has lost PGRS at a faster rate than poplar after their divergence.

### Compare the ω ratios for PGRS within and between the two lineages

The Ka, Ks, and ω ratios were calculated for each PGRS on the 19 chromosomes in poplar and willow separately. At the genome-wide level, the average ω ratio for *P. trichocarpa* and *S. suchowensis* was 0.309 and 0.274, respectively (Table 1). The former was significantly higher than that for the latter (*P* < 0.001) (Table 2), indicating PGRS in willow were subject to stronger purifying selection than those in poplar, which would result in a faster loss of redundant genes in willow than in poplar. At chromosome level, the average ω ratios for PGRS on different chromosomes ranged from 0.301 to 0.318 in poplar (Table 1), and it ranged from 0.261 to 0.282 in willow (Table 1). The ω ratios were all substantially smaller than 1, indicating PGRS were generally under strong purifying selection in both lineages. The statistical test for ω ratios of PGRS between willow and poplar indicated that this parameter varied significantly among 18 of the corresponding chromosomes except for chromosome XVIII (Table 2), suggesting that PGRS on most of the chromosomes in willow were under significantly stronger purifying selection than those on the corresponding chromosomes in poplar. In contrast, no significant difference was observed with ω ratios for most of the pairwise comparisons within the genome of poplar (except for XIX vs. IV, XIX vs. VI, and XIX vs. IX) and willow (except for IV vs. V) (Table 3; Table 4), indicating that PGRS on most chromosomes within each lineage were under similar purifying selection pressure.

**Table 2:** Statistical test for ω ratios of PGRS on the corresponding chromosomes between *P. trichocarpa* and *S. suchowenesis*

*Salix*	*Populus*	*P*-value
I	I	0.0000^a^
II	II	0.0000^a^
III	III	0.0046^a^
IV	IV	0.0000^a^
V	V	0.0057^a^
VI	VI	0.0001^a^
VII	VII	0.0468^a^
VIII	VIII	0.0001^a^
IX	IX	0.0002^a^
X	X	0.0001^a^
XI	XI	0.0004^a^
XII	XII	0.0115^a^
XIII	XIII	0.0051^a^
XIV	XIV	0.0001^a^
XV	XV	0.0017^a^
XVI	XVI	0.0161^a^
XVII	XVII	0.0002^a^
XVIII	XVIII	0.0535
XIX	XIX	0.0001^a^
SDR	SDR	0.5835
Genome wide	Genome wide	0.0000^a^

^a^indicate significance at *P* ≤ 0.05

**Table 3:** Statistical test for ω ratios of PGRS among chromosomes within the genome of *P. trichocarpa*

Chromosome	II	III	IV	V	VI	VII	VIII	IX	X	XI	XII	XIII	XIV	XV	XVI	XVII	XVIII	XIX	SDR	Genome wide
I	0.840	0.450	0.264	0.533	0.307	0.667	0.866	0.236	0.866	0.422	0.636	0.682	0.844	0.362	0.439	0.474	0.450	0.169	0.410	0.681
II		0.375	0.218	0.451	0.250	0.592	0.742	0.202	0.742	0.531	0.559	0.801	0.963	0.318	0.356	0.609	0.387	0.224	0.403	0.553
III			0.757	0.855	0.841	0.830	0.592	0.669	0.592	0.177	0.867	0.337	0.433	0.816	0.896	0.235	0.901	0.073	0.503	0.559
IV				0.601	0.901	0.632	0.391	0.914	0.391	0.105	0.641	0.215	0.280	0.952	0.900	0.146	0.870	0.040^a^	0.533	0.320
V					0.688	0.941	0.684	0.544	0.684	0.208	0.975	0.386	0.491	0.696	0.758	0.271	0.790	0.081	0.467	0.689
VI						0.700	0.454	0.808	0.454	0.124	0.734	0.246	0.312	0.941	0.987	0.164	0.946	0.050^a^	0.554	0.367
VII							0.791	0.584	0.791	0.303	0.981	0.488	0.601	0.696	0.751	0.352	0.778	0.127	0.445	0.827
VIII								0.350	1.000	0.380	0.761	0.599	0.748	0.478	0.547	0.431	0.587	0.154	0.420	0.900
IX									0.350	0.096	0.584	0.195	0.256	0.871	0.822	0.142	0.792	0.039^a^	0.570	0.285
X										0.380	0.761	0.599	0.748	0.478	0.547	0.431	0.587	0.154	0.420	0.900
XI											0.292	0.749	0.594	0.153	0.195	0.963	0.198	0.515	0.291	0.243
XII												0.463	0.578	0.716	0.784	0.332	0.807	0.121	0.456	0.792
XIII													0.852	0.271	0.327	0.778	0.329	0.364	0.349	0.477
XIV														0.355	0.392	0.662	0.424	0.278	0.399	0.616
XV															0.931	0.190	0.913	0.060	0.511	0.450
XVI																0.227	0.961	0.083	0.556	0.523
XVII																	0.240	0.542	0.336	0.316
XVIII																		0.080	0.552	0.557
XIX																			0.230	0.092
SDR																				0.436

^a^indicate significance at *P* ≤ 0.05

**Table 4:** Statistical test for ω ratios of PGRS among chromosomes within the genome of *S. suchowensis*

Chromosome	II	III	IV	V	VI	VII	VIII	IX	X	XI	XII	XIII	XIV	XV	XVI	XVII	XVIII	XIX	SDR	Genome wide
I	0.920	0.565	0.203	0.275	0.701	0.472	0.780	0.326	0.780	0.760	0.975	0.552	0.483	0.500	0.565	0.509	0.747	0.541	0.463	0.995
II		0.561	0.258	0.263	0.790	0.438	0.717	0.409	0.717	0.712	0.935	0.523	0.572	0.545	0.561	0.546	0.676	0.572	0.493	0.904
III			0.097	0.629	0.387	0.774	0.782	0.165	0.782	0.902	0.705	0.879	0.283	0.294	1.000	0.338	0.960	0.361	0.420	0.504
IV				0.027^a^	0.392	0.095	0.139	0.833	0.139	0.195	0.330	0.115	0.688	0.691	0.097	0.839	0.219	0.796	0.601	0.124
V					0.167	0.951	0.443	0.061	0.443	0.603	0.462	0.811	0.136	0.135	0.629	0.166	0.659	0.191	0.377	0.176
VI						0.331	0.532	0.555	0.532	0.556	0.752	0.384	0.712	0.735	0.387	0.698	0.557	0.730	0.501	0.633
VII							0.608	0.155	0.608	0.707	0.561	0.868	0.256	0.224	0.774	0.260	0.752	0.274	0.383	0.412
VIII								0.226	1.000	0.893	0.884	0.703	0.376	0.414	0.782	0.398	0.896	0.442	0.458	0.734
IX									0.226	0.283	0.428	0.185	0.869	0.833	0.165	0.972	0.317	0.935	0.579	0.246
X										0.893	0.884	0.703	0.376	0.414	0.782	0.398	0.896	0.442	0.458	0.734
XI											0.804	0.813	0.423	0.387	0.902	0.413	0.970	0.441	0.415	0.718
XII												0.634	0.599	0.615	0.705	0.602	0.790	0.599	0.472	0.980
XIII													0.307	0.271	0.879	0.314	0.863	0.329	0.393	0.502
XIV														0.939	0.283	0.910	0.407	0.909	0.558	0.437
XV															0.294	0.963	0.385	0.974	0.569	0.440
XVI																0.338	0.960	0.361	0.420	0.504
XVII																	0.416	0.990	0.601	0.461
XVIII																		0.428	0.419	0.704
XIX																			0.586	0.503
SDR																				0.469

^a^indicate significance at *P* ≤ 0.05

Examination of the selection pressure on genes in the sex-determining region will provide unique insight into the evolution of sex chromosomes. Previous studies have shown that the gender locus in *Populus* was mapped to the peritelomeric region upstream to the position of SSR marker O_206 in chromosome XIX^22,33^. The gender locus in *Salix* was between SSR markers SSR151 and SSR893 on chromosome XV^23^. In this study, ω ratios were calculated for the PGRS in SDRs for each lineage. The median ω ratios were 0.233 and 0.223 in the SDR of the *P. trichocarpa* and *S*. *suchowensis* genomes, respectively, which is the lowest value in the corresponding column (Table 1). Thus, higher convergent selection pressure has been observed to act on the PGRS in SDRs, and PGRS in the corresponding regions are supposed to be lost faster. Interestingly, much lower PGRS density was observed in the SDRs in both the *P. trichocarpa* (11.9/Mb) and *S*. *suchowensis* (14.9/Mb) genomes (Table 1). It is well known that gene losses occur with the evolution of sex chromosomes. A dramatic decrease in PGRS density in SDR regions indicates the faster divergence of SDRs in the two lineages.

### Sliding window analysis

**Fig. 2:**
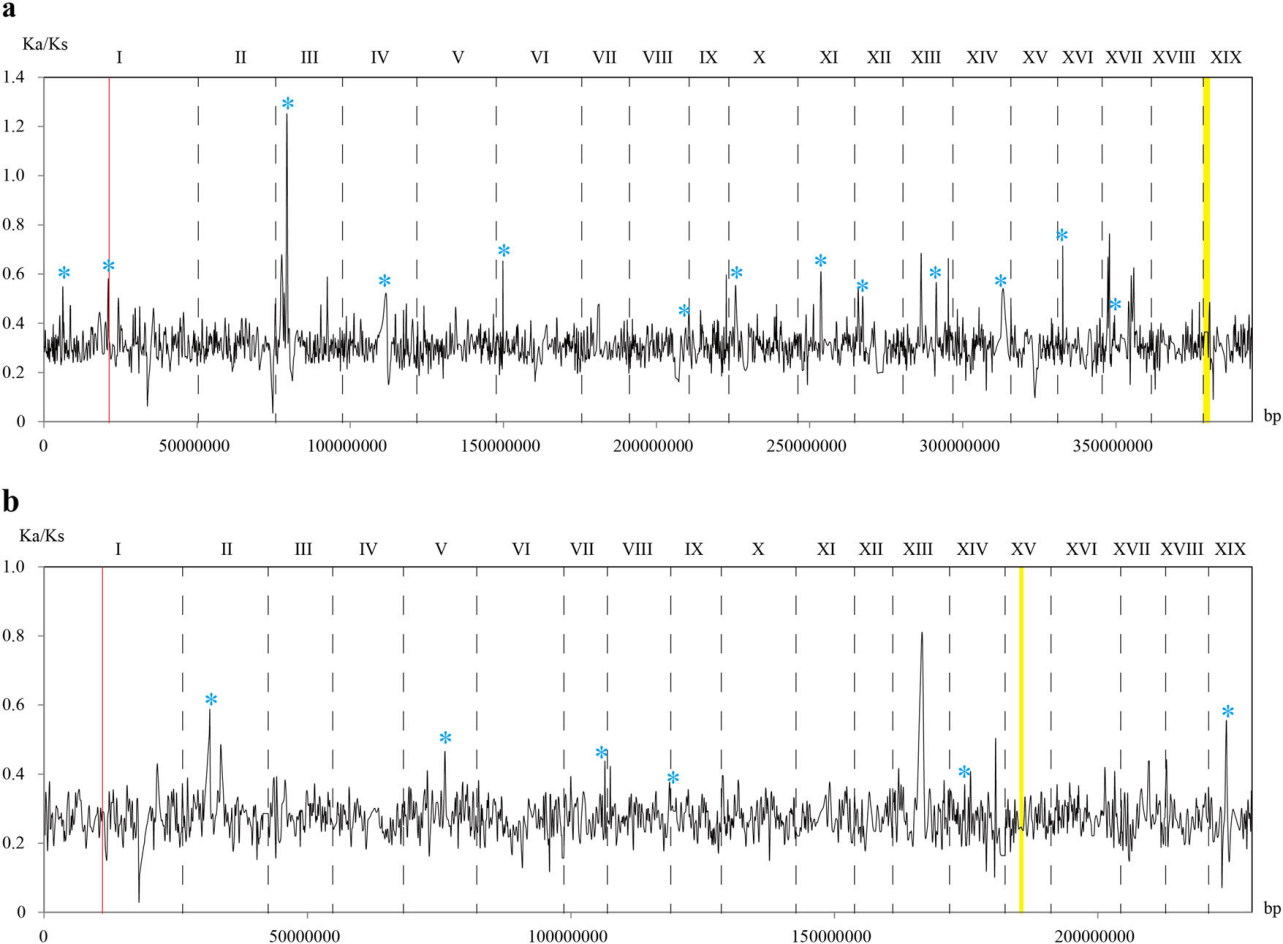
Sliding window analysis of ω ratios varying along each chromosome in *P*. *trichocarpa* and *S. suchowensis* **a** Variation of ω ratios along chromosomes in *P*. t*richocarpa*. **b** Variation of ω ratios along chromosomes in *S*. *suchowensis*. Note: blue stars indicate the regions where ω ratios varied significantly in the corresponding regions between *P*. *trichocarpa and S. suchowensis*; red lines represent the genomic regions where fission and fusion occurred on chromosome I and chromosome XVI; yellow regions represent the SDRs

To demonstrate the variation of selection pressure along each chromosome, we conducted sliding window analysis for poplar (Fig. 2a) and willow (Fig. 2b) separately. The figures show that extensive purifying selection dominated throughout each chromosome. Genome regions under significant relaxed purifying selection were observed on many of the chromosomes (peak positions). Examination of the sliding windows detected significantly elevated ω ratios in 13 regions on 12 of the chromosomes in poplar and in six regions on six of the chromosomes in willow. A detailed examination of ω ratios revealed 25 PGRS that were subjected to extremely strong purifying selection (ω = 0) and 52 PGRS were under positive selection (ω > 1) (Supplementary Table S3) in the *P. trichocarpa* genome, accounting for 0.28% and 0.58% of the total, respectively. In the willow genome, the PGRS under unusually strong purifying (ω = 0) and positive selection (ω > 1) were 8 and 3, (Supplementary Table S3), accounting for 0.16% and 0.06% of the total, respectively. It is noteworthy that in both lineages, PGRS under extremely strong purifying selection are mainly housekeeping genes coding histone, ubiquitin and ribosomal proteins. In contrast, GO enrichment showed that PGRS under positive selection were significantly enriched in genes found with the “metabolic process” terms associated with a diverse spectrum of biological functions (Supplementary Fig. S3), especially genes involving tolerance to biotic or abiotic stress, such as the bifunctional inhibitor, BTB/POZ domain-containing protein, and the AWPM-19-like family protein, etc. In the willow genome, the three PGRS under positive selection were annotated as genes with unknown function; thus, it remained unclear which biological processes they might be involved with.

## Discussion

Poplar and willow are dioecious woody plants that generally appear as diploids with a basic haploid chromosome number of *n* = 19. It has been confirmed that *Populus* and *Salix* share lineage-specific salicoid duplication, and nearly every chromosomal segment is found to have a paralogous segment elsewhere in their genomes^14,15,26^. Collinearity analysis of genetic maps and genome sequences for multiple Salicaceae species showed that poplar and willow shared the same large-scale genomic history^16,21,34,35^. Analyzing the orthologous groups suggested that the divergence of these two lineages occurred approximately 6 million years later after salicoid duplication^15^. However, it remains unknown whether, after salicoid duplication, fission and fusion of the ancestral chromosomes first gave rise to the crown of *Populus* or that of *Salix*. Previous studies revealed that chromosome I of poplar or willow was a conjunction of chromosome XVI and the distal end of chromosome I of the alternate lineage, and the proximal end of chromosome I in poplar or willow corresponded to chromosome XVI of the alternate lineage. These major interchromosomal rearrangements distinguish the karyotypes of poplars and willows^16,21^. The changes accumulated during speciation may be of special relevance in understanding the basis of their differences. In this study, significantly elevated ω ratios were observed in different regions on many of the chromosomes. The elevated ω ratio means relaxed purifying selection; thus, the corresponding genomic regions are supposed to diverge faster. In the poplar genome, significantly elevated ω ratios were observed on chromosome I and XVI, where chromosomal fission and fusion occurred. In contrast, no peaks appeared in the corresponding regions in the willow genome. Whether the observed coincidences are relevant to the additional round of chromosome rearrangements is an interesting question. However, with the current data, we cannot determine whether the observed genome regions with significantly elevated ω ratios are stable signatures and this needs to be explored in more species.

In this study, we also found that the PGRS in willow were subject to stronger purifying selection than those in poplar, which would result in a faster loss of PGRS in willow. Deleterious mutations are much more likely to occur than beneficial ones. Thus, the paralogous copies of a gene may often accumulate degenerative mutations at an accelerated rate following a duplication event, and purifying selection can result in stabilizing selection through the purging of deleterious variations that arise^36^. We speculate that the mechanism underlying the faster loss of PGRS in willow might relate to the additional round of genome reorganization after salicoid duplication, since chromosomal rearrangements might bring up epistatic effects on other chromosome regions and affect genome stability. During genome stabilization, the genome of the nascent lineage would reshuffle more extensively. During this process, good genes might be dragged off due to the hijacking effect, which would cause a driven force for more active gene duplication through other manners in willow. Indeed, significantly more active gene duplications associated with transposon and tandem duplication were detected in willow than in poplar^15^. The joint driving force would cause willow to evolve faster, leading willows to be more diverse. According to the taxonomy of the Salicaceae family, the genus *Populus* comprises 29 species or so^37^, while the genus *Salix* represents over 300–500 species^38,39^. Moreover, *Salix* shows considerable variation in size, growth form and crown architecture, ranging from large trees to sub-trees to dwarf shrubs, while *Populus* generally appear as large trees. Taking these findings together, we propose that *Populus* should be evolutionarily more primitive than *Salix*, which supports the empirical presumption in previous studies^13,39,40^.

It was found that different autosomes evolved into sex chromosomes in the sister genera of *Populus* and *Salix*^21,23^. In *Populus*, the gender locus was consistently mapped on chromosome XIX^22,33,41–43^, and multiple lines of evidence suggest that chromosome XIX has been evolving into an incipient sex chromosome^44,45^, while chromosome XIX is an autosome in willow. In *Salix*, the primitive sex chromosome is chromosome XV^21,23^, while the corresponding chromosome is an autosome in poplar. Examination of the SDRs revealed stronger purification selection and faster loss of PGRS both in poplar and willow. At the chromosome level, chromosome XIX is characterized by the lowest gene and PGRS densities in both lineages, but this characteristic is not observed on chromosome XV. It is a common scenario that sex chromosomes contain less genes than autosomes in dioecious organisms^46^. We proposed that *Populus* might inherit the ancestral sex chromosome from the progenitor of the Salicaceae family, and the sex chromosome in willow should be evolutionarily younger. Evidence in this study showed that the sex chromosome in willow was still at the very early evolutionary stage because dramatic loss of PGRS was observed only in its SDR but not at the chromosomal level on chromosome XV.

It has been reported that retaining genes should be biased after WGD^47,48^. In *Brassica* species, asymmetrical gene retention was proposed to contribute to extreme morphological diversity^49,50^. It is commonly accepted that the rate of molecular evolution differs greatly from gene to gene depending on the degree of constraint of gene products^51^. In this study, we detected some genes under extremely strong purifying selection or under positive selection in both lineages. As expected by the neutral theory of molecular evolution^51^, these genes should account for only a very small portion of the total. Genes under extremely strong purifying selection (ω = 0) are mainly housekeeping genes, are subject to very stringently selective constraints, and every nonsynonymous mutation in them is supposed to be deleterious. By contrast, PGRS under positive selection (ω > 1) are assumed to aid adaption and fitness, and they are found to be mainly involved in transcriptional regulation and resistance to biotic or abiotic stresses. These genes tend to diverge faster to gain better fitness for the population. Thus, from a biological perspective, genes under unusual selection pressure detected in this study are worthy of further functional exploration through experiments.

## Materials and methods

### Genome sequence data

Whole-genome CDS sequences, protein sequences and gene positions along each chromosome in the genome of *P. trichocarpa* were extracted from the Joint Genome Institute, United States Department of Energy website (JGI) (https://genome.jgi.doe.gov/portal/pages/dynamicOrganismDownload.jsf?organism = Ptrichocarpa). The corresponding information for *S. suchowensis* was retrieved from willow genome databases (http://115.29.234.170/node/5). If a gene had more than one transcript, only the first transcript in the annotation was extracted.

### Detection of PGRS in *P. trichocarpa* and *S. suchowensis*

The whole-genome protein sequences from *P. trichocarpa* and *S. suchowensis* were compared against themselves using BLASTP to search for their paralogs^52^. For a protein sequence, the best five non-self hits in each genome were reported with an E-value threshold of 10^−10^. Whole-genome duplication, tandem gene duplication, and segmental duplication all generate paralogous genes^3^. To detect the paralogous genes specifically generated by the salicoid duplication event, we first identified the syntenic segments containing at least five homologous genes that are collinear in a row, following the description in Tang et al.’s paper^53^. In detail, whole-genome BLASTP results were sorted according to gene positions in poplar and willow genomes. Then, the sorted paralogs were used to compute collinear blocks for all chromosomes to detect the WGD paralogous genes using MCScanX^54^. For each pair of paralogous duplicates, their protein sequences were aligned using MUSCLE^55^, and the protein alignment was converted to DNA alignment using PAL2NAL according to their CDS sequences^56^. Subsequently, we confined the paralogous genes associated with salicoid duplication by calculating the average 4DTV for each syntenic segment with all the contained paralogous genes, and 4DTV values for the paralogous genes having ≥10 four-fold degenerate sites were calculated using in-house Perl scripts following Rodgers-Melnick et al.’s study^32^. The 4DTV range associated with salicoid duplication was determined by plotting the 4DTV values. Ka, Ks values for the paralogous genes were calculated using the Nei-Gojobori algorithm implemented in the KaKs_Calculator 2.0^57^. PGRS were finally determined based on the plotting of the Ks values in each lineage. The 4DTV and Ks ranges were set to cover 99% of the variables for the peak associated with salicoid duplication.

### Significance test for ω ratios and sliding window analysis

A significance test for ω ratios was performed with Mann-Whitney statistics to detect whether there is significantly different selection pressure acting on specific genomic regions within or between poplar and willow, with a significance level of *P* ≤ 0.05. The Mann-Whitney test is particularly useful for determining whether there is a significant difference for two groups of samples with unequal sizes based upon a series of ranking scores^58^. This test was conducted with Minitab software (Minitab Inc., PA, USA), and ω ratios were transformed into ranking scores by the software prior to the test. To compare the detailed patterns of selection pressure acting on different chromosomes between the two lineages, we open a sliding window along each chromosome. Because willow had a smaller gene content than poplar, a sliding window was designed to contain 30 and 25 genes in poplar and willow, respectively. The default sliding size was 15-gene lengths.

For the ω indicator, ω > 1 indicates positive selection, ω close to 1 indicates neutral mutation, and ω < 1 indicates purifying (negative) selection^29^. We detected segmental duplicates under unusual selection pressure in poplar and willow, with ω > 1 and ω = 0, and annotated these genes by referring annotation files in the JGI and willow genome databases, respectively. GO-based functional enrichment analysis was performed for the genes under positive selection using Blast2GO (https://www.blast2go.com/).

## Supplementary information

Synteny of PGRS among the 19 chromosomes in the genome of *P. trichocarpa*

Synteny of PGRS among the 19 chromosomes in the genome of *S. suchowensis*

GO enrichment of PGRS under positive selection in the genome of *P. trichocarpa*

The Ks and ω ratios for PGRS in the genome of *P. trichocarpa*

The Ks and ω ratios for PGRS in the genome of *S. suchowensis*

The PGRS under unusual selection pressure in the genome of *P. trichocarpa*and *S. suchowensis*

Supplementary information
