# Supplementary figures and images for: Uneven selection pressure accelerating divergence of Populus and Salix
Source: Hortic Res. 2019 Apr 6;6:37. doi: 10.1038/s41438-019-0121-y (PMC6450953; doi:10.1038/s41438-019-0121-y)

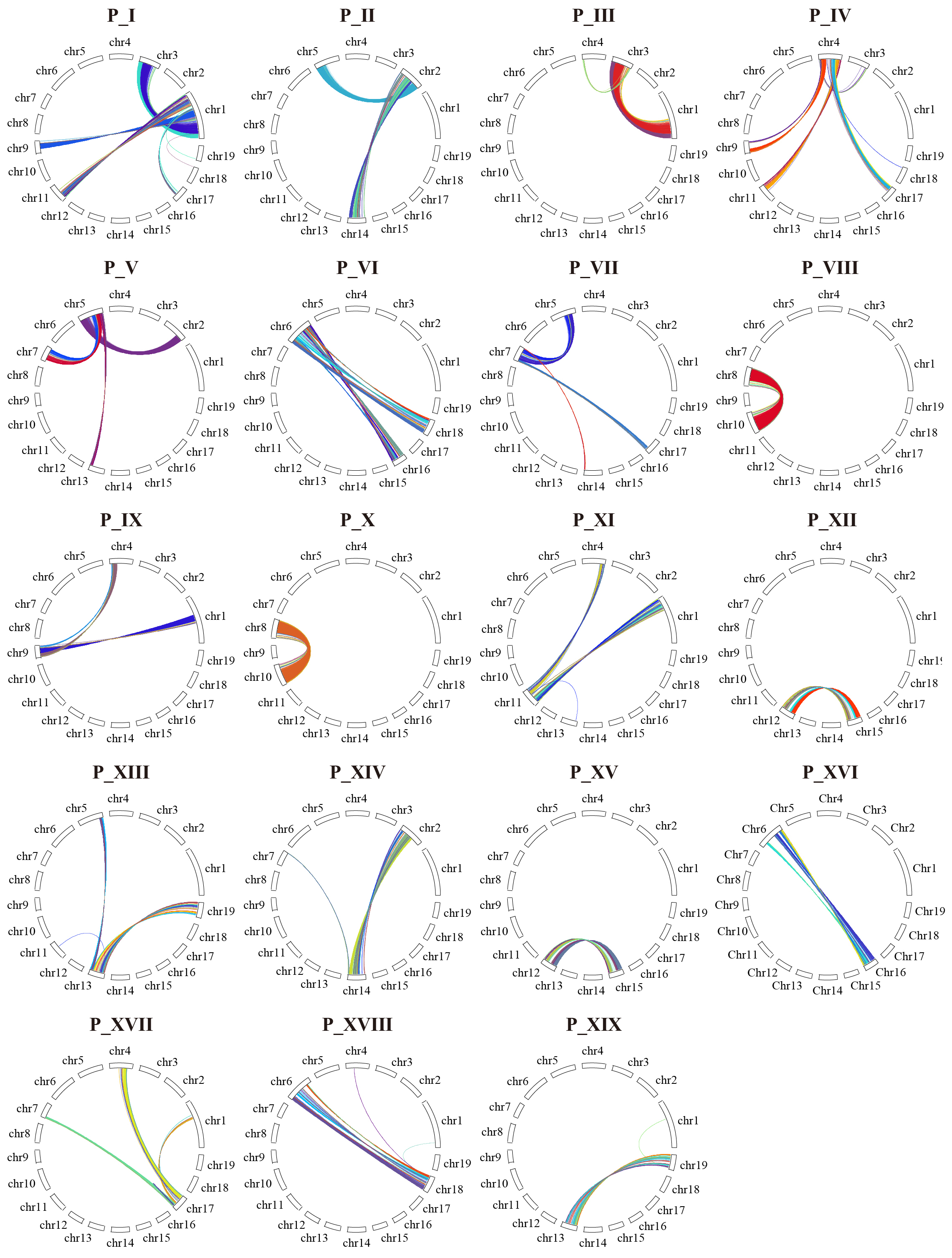

Supplement: Supplementary file 1 — Synteny of PGRS among the 19 chromosomes in the genome of P. trichocarpa [file 41438_2019_121_MOESM1_ESM.jpg]

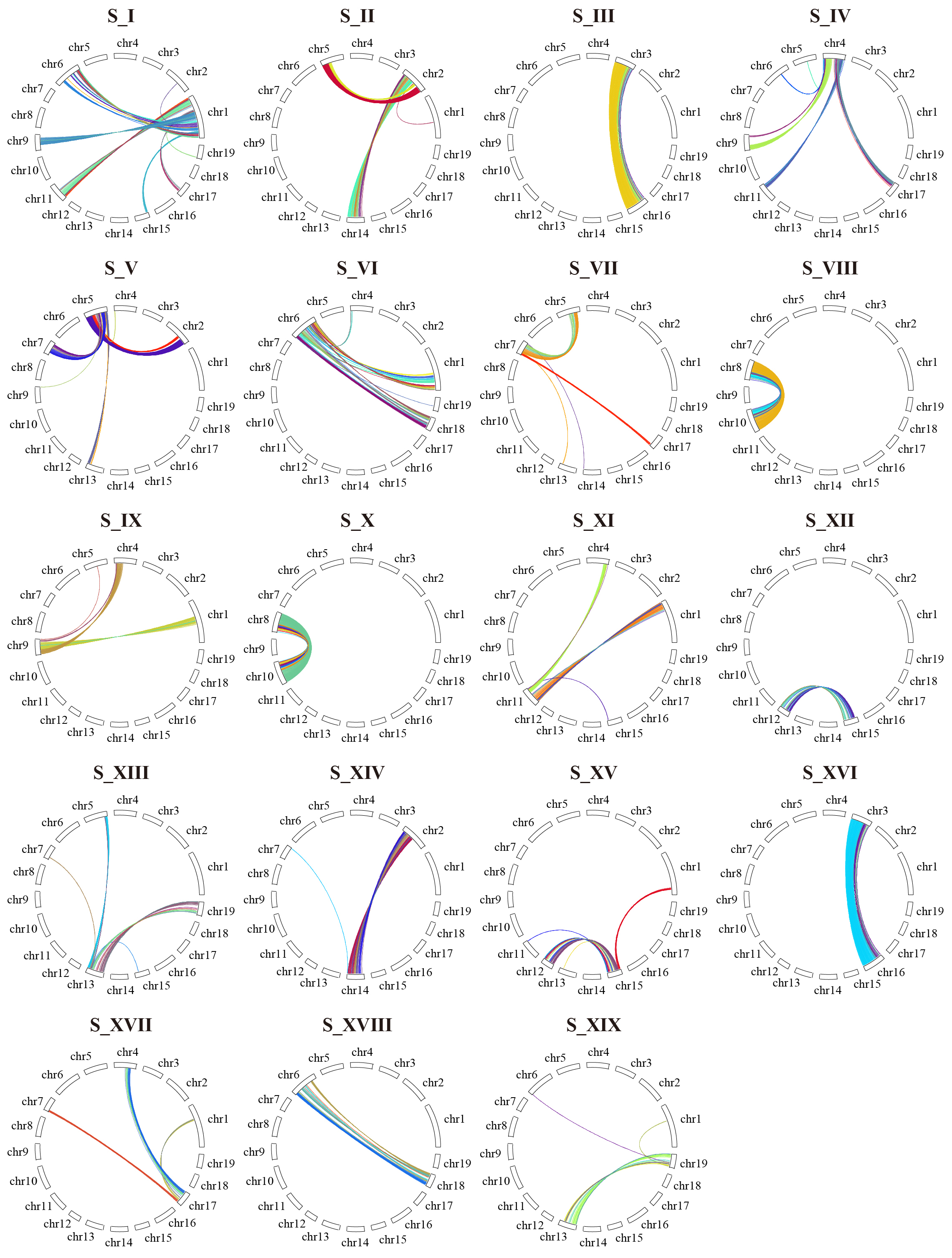

Supplement: Supplementary file 2 — Synteny of PGRS among the 19 chromosomes in the genome of S. suchowensis [file 41438_2019_121_MOESM2_ESM.jpg]

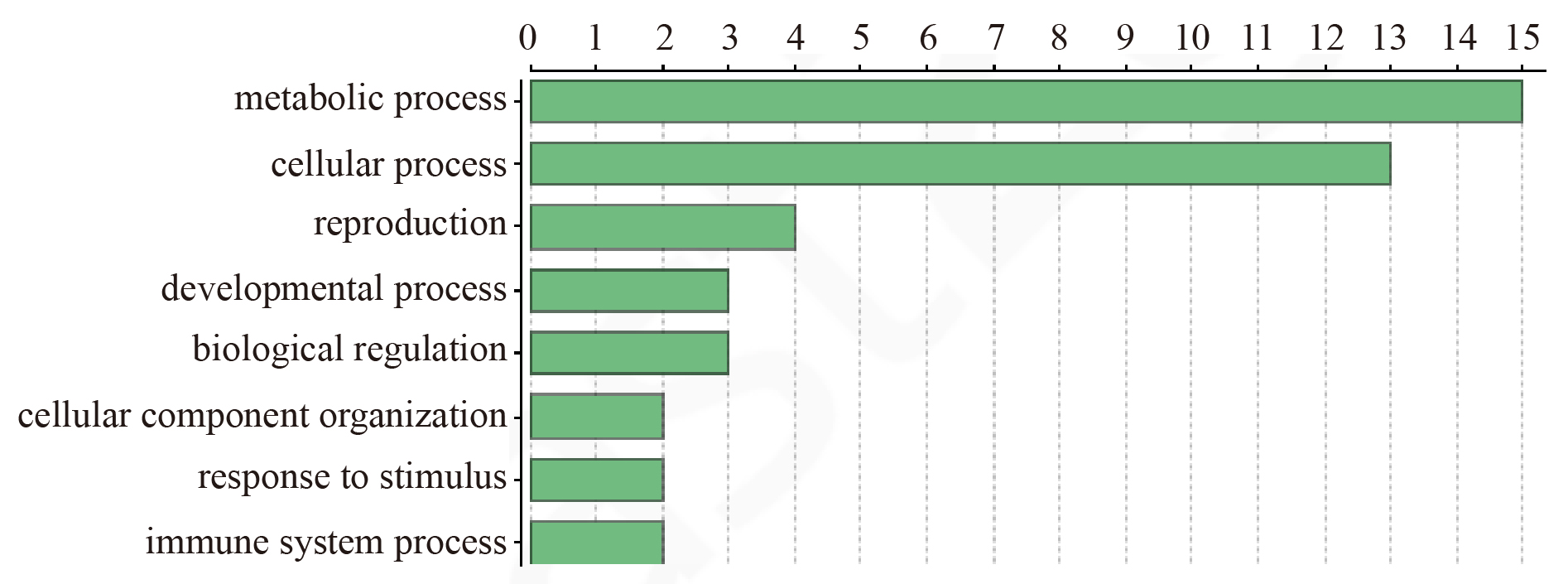

Supplement: Supplementary file 3 — GO enrichment of PGRS under positive selection in the genome of P. trichocarpa [file 41438_2019_121_MOESM3_ESM.jpg]
