# Supplementary material for: Uneven selection pressure accelerating divergence of Populus and Salix
Source: Hortic Res. 2019 Apr 6;6:37. doi: 10.1038/s41438-019-0121-y (PMC6450953; doi:10.1038/s41438-019-0121-y)
Supplement: Supplementary file 7 — Supplementary information [file 41438_2019_121_MOESM7_ESM.docx]

**Supplementary Material**

**Supplementary Table S1** The Ks and ω ratios for PGRS in the genome of *P. trichocarpa*

**Supplementary Table S2** The Ks and ω ratios for PGRS in the genome of *S. suchowensis*

**Supplementary Table S3** The PGRS under unusual selection pressure in the genome of *P. trichocarpa* and *S. suchowensis*

**Supplementary Fig. S1** Synteny of PGRS among the 19 chromosomes in the genome of *P. trichocarpa*

**Supplementary Fig. S2** Synteny of PGRS among the 19 chromosomes in the genome of *S. suchowensis*

**Supplementary Fig. S3** GO enrichment of PGRS under positive selection in the genome of
